# [ _18_ F]PSMA Tracer and _131_ I Avid Retroperitoneal Thyroid Tissue in a Patient with Synchronous Thyroid and Prostate Carcinomas: First Case Report and Literature Review

**DOI:** 10.1055/s-0045-1809147

**Published:** 2025-05-13

**Authors:** Chamani Punchihewa, Juliette Zeilmaker, Maged Elsewafy, Sabina Dizdarevic

**Affiliations:** 1 Department of Nuclear Medicine, University Hospitals Sussex NHS Foundation Trust, Hove, United Kingdom; 2 Life Sciences and Medicine, King's College London, London, United Kingdom

**Keywords:** PSMA-PET-CT, synchronous malignancies, thyroid cancer, prostate cancer, retroperitoneal metastasis, diagnostic pitfalls

## Abstract

We present the first described case of retroperitoneal metastasis from follicular thyroid carcinoma (FTC). This was incidentally discovered as a PSMA (prostate-specific membrane antigen)-positive lesion on PSMA-positron emission tomography (PET)-computed tomography (CT) in a patient with synchronous prostate cancer (PCa).The expanding utilization of PSMA-PET-CT has revealed tracer uptake in several nonprostatic conditions. A 68-year-old man investigated for PCa, underwent magnetic resonance imaging, which revealed an 18-mm retroperitoneal soft tissue nodule lateral to the left psoas and a left pelvic node. PSMA-PET-CT showed tracer uptake in the primary PCa, retroperitoneal lesion, and pelvic node with an incidental high-grade focus in the thyroid. A CT following a period of androgen deprivation demonstrated no response in the retroperitoneal lesion, while the pelvic node became smaller. Fine-needle aspiration (FNA) of the thyroid was performed, although an ultrasound was initially reported as benign. FNA cytology (FNAC) was interpreted as a benign nodule. However, CT-guided biopsy of the retroperitoneal lesion revealed follicular thyroid tissue. The differential diagnoses were ectopic thyroid tissue and FTC. FNAC and ultrasound were reviewed at the thyroid multidisciplinary meeting (MDM) and upgraded to follicular atypia and suspicious for malignancy, respectively. Left hemithyroidectomy confirmed an angioinvasive follicular carcinoma. Completion thyroidectomy revealed a small incidental micropapillary carcinoma. Single photon emission computed tomography (SPECT)-CT post- ^131^ I treatment showed intensely iodine-avid tissue within the thyroid bed and retroperitoneal deposit. On follow-up ^123^ I-SPECT-CTs, there was no abnormal iodine uptake and the retroperitoneal deposit decreased from 18 to 5 mm, presumed as scar tissue. Thyroglobulin reduced from 7.7 to < 0.1 ug/L. MDM recommended 6 monthly surveillance. PSMA-positive lesion evaluation can be challenging due to PSMA expression in nonprostatic conditions. As illustrated by this case, unusual distribution of tracer uptake requires further investigations and a multidisciplinary approach to guide management. High PSMA expression in differentiated thyroid cancer was associated with shorter progression-free survival and may be considered a marker of aggressiveness. Such tumors could be candidates for targeted PSMA-radioligand therapy (e.g., ^177^ lutetium), particularly in radioiodine-negative/refractory cases, which are difficult to treat.

## Introduction

We present a unique case of a solitary retroperitoneal metastasis from follicular thyroid carcinoma (FTC), incidentally detected by a staging [18F]-prostate-specific membrane antigen (PSMA)-1007-positron emission tomography (PET)-computed tomography (CT) for prostate cancer (PCa) and confirmed by subsequent investigations. Although various other metastatic sites of FTC have been reported, there were no previous cases of retroperitoneal metastasis of FTC reported in the literature.

PSMA is a protein found in healthy prostate cells but overexpressed 100- to 1,000-fold in PCa cells. The expanding utilization of PSMA-PET-CT has revealed PSMA-ligand uptake in other tissues/conditions, including normal nonprostatic epithelial cells, inflammation/infection, nonprostatic neoplastic cells, and tumor-associated neovasculature. 1 As exemplified by this case, it is important to investigate unusual sites of uptake on PSMA-PET-CT to exclude synchronous tumors.

PSMA-PET-CT is utilized for detection, staging, and response assessment in PCa and has paved the way for theragnostic applications. Hence, other malignancies such as thyroid cancer with PSMA expression could potentially be susceptible to PSMA-targeted therapy, particularly if they are radioiodine-resistant.

## Case Report

A 68-year-old male was investigated for hematuria. Prostate-specific antigen (PSA) was elevated at 12 ng/mL. Magnetic resonance imaging (MRI) showed bilateral PI-RADS 5 (Prostate Imaging Reporting and Data System) lesions in the prostate, a left 18-mm retroperitoneal soft tissue nodule lateral to the psoas, and an 8-mm left pelvic node.

**Fig. 1:**
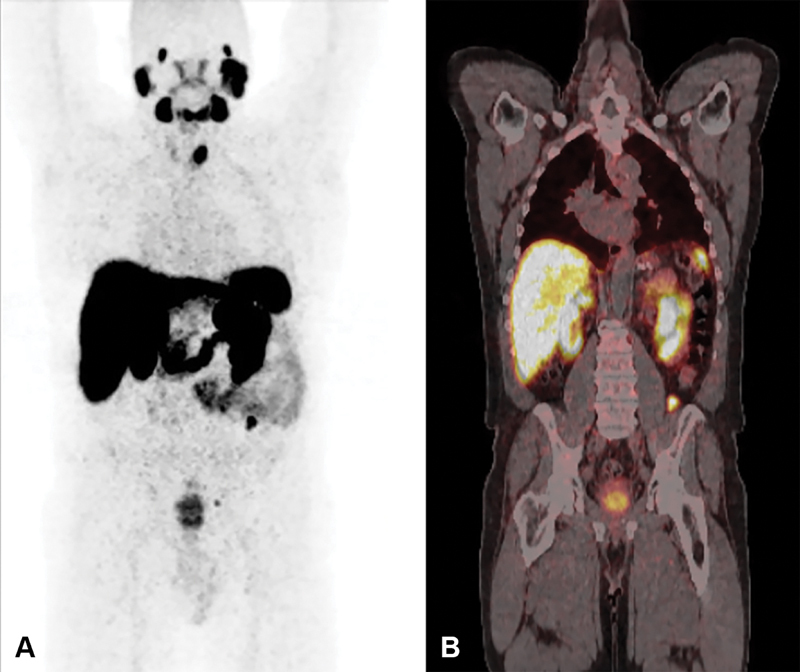
( **A** , **B** ) Prostate-specific membrane antigen (PSMA)-positron emission tomography (PET)-computed tomography (CT) (coronal) images showing intense focal uptake in the prostate, small left iliac node, 18 mm left of the retroperitoneal lesion, and in a left thyroid nodule.

**Fig. 2:**
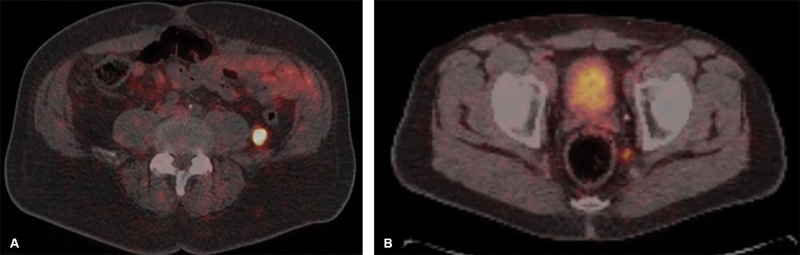
( **A** , **B** ) Intensely tracer-avid left retroperitoneal lesion and left pelvic node on axial prostate-specific membrane antigen (PSMA)-positron emission tomography (PET)-computed tomography (CT) images.

**Fig. 3:**
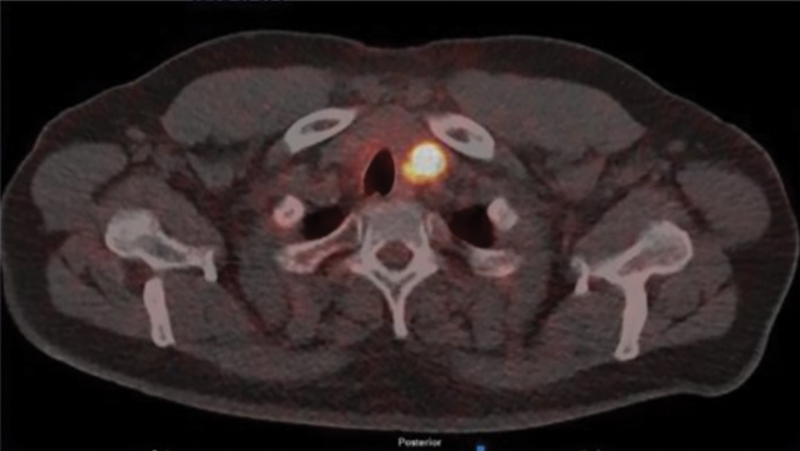
Axial prostate-specific membrane antigen (PSMA)-positron emission tomography (PET)-computed tomography (CT) images showing high-grade uptake in the left thyroid nodule.

**Fig. 4:**
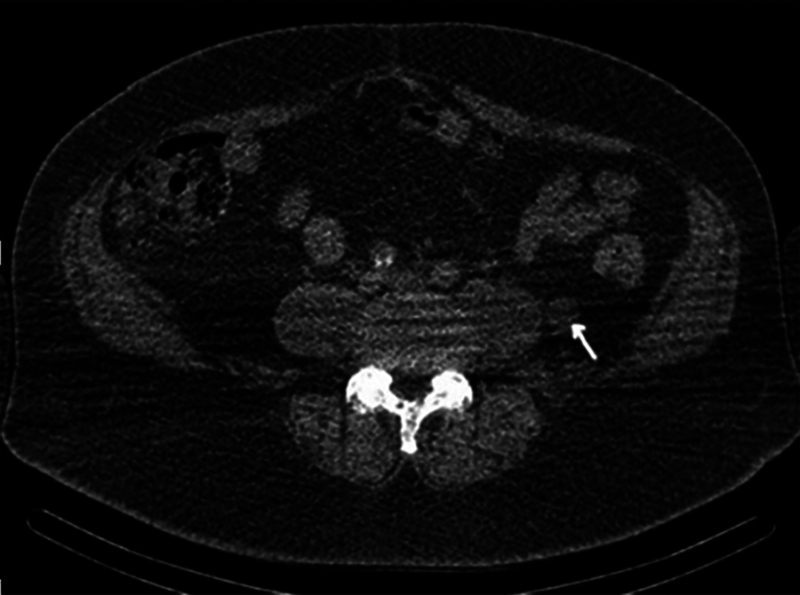
Computed tomography (CT) following a period of androgen deprivation demonstrated no response in the retroperitoneal lesion.

The patient underwent PSMA-PET-CT for staging. As shown in Fig. 1 , there was intense PSMA tracer uptake in a primary PCa as well as in the retroperitoneal lesion and left pelvic node, which are further demonstrated on axial images ( Fig. 2 ). There was an incidental high-grade focus of uptake in the left thyroid lobe as shown in Fig. 3 . Following a period of androgen deprivation therapy for PCa, CT ( Fig. 4 ) demonstrated no response in the retroperitoneal lesion, while the pelvic node decreased from 8 to 5 mm. Although initially thought to represent metastasis from PCa, the atypical retroperitoneal location of the lesion, lack of response to treatment, and incidental PSMA-tracer uptake in the thyroid prompted further investigations to rule out a synchronous primary.

An initial thyroid ultrasound (US) was reported as benign (U2 nodule), 2 yet fine-needle aspiration (FNA) of the PSMA-positive thyroid nodule was performed nevertheless. Cytology was interpreted initially as a benign nodule (THY-2). 3 However, CT-guided biopsy of the retroperitoneal lesion revealed follicular thyroid tissue with no papillary features. Immunohistochemistry confirmed that a prostatic marker NKX3.1 (homeodomain-containing transcription factor) was negative. The differential diagnoses were between metastatic thyroid follicular carcinoma and ectopic retroperitoneal thyroid tissue. However, as normal thyroid tissue is not PSMA-tracer avid and there was also an intensely PSMA-positive thyroid nodule, FNA cytology (FNAC) and US were reviewed at the thyroid multidisciplinary meeting (MDM). Findings were upgraded to follicular atypia (Thy3a) and suspicious for malignancy (U4), respectively. This warranted a left hemithyroidectomy, which confirmed a 33-mm angioinvasive follicular carcinoma. MDM discussion was pivotal in this case by enhancing US and FNAC findings and directing surgical intervention. Completion thyroidectomy revealed a small incidental micropapillary carcinoma, unrelated to the known retroperitoneal disease.

**Fig. 5:**
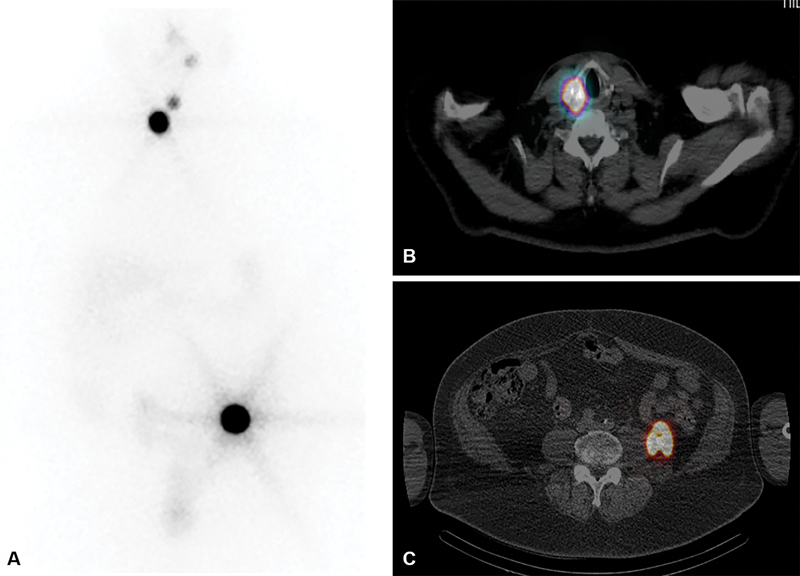
( **A** – **C** ) Single photon emission computed tomography (SPECT)-computed tomography (CT) post-first ^131^ I treatment showing intense iodine avid tissue in the thyroid bed and in the retroperitoneal deposit.

Postoperatively, the patient received 3791 MBq of ^131^ I as first radioactive iodine treatment (RAIT) for thyroid carcinoma. Single photon emission computed tomography-CT (SPECT-CT) a few days after the first RAIT ( Fig. 5 ) showed high-grade iodine-avid tissue in the thyroid bed and within the retroperitoneal deposit. Thyroglobulin was elevated (7.7 ug/L) and thyroglobulin antibodies were also high (5.3 kU/L) initially.

**Fig. 6:**
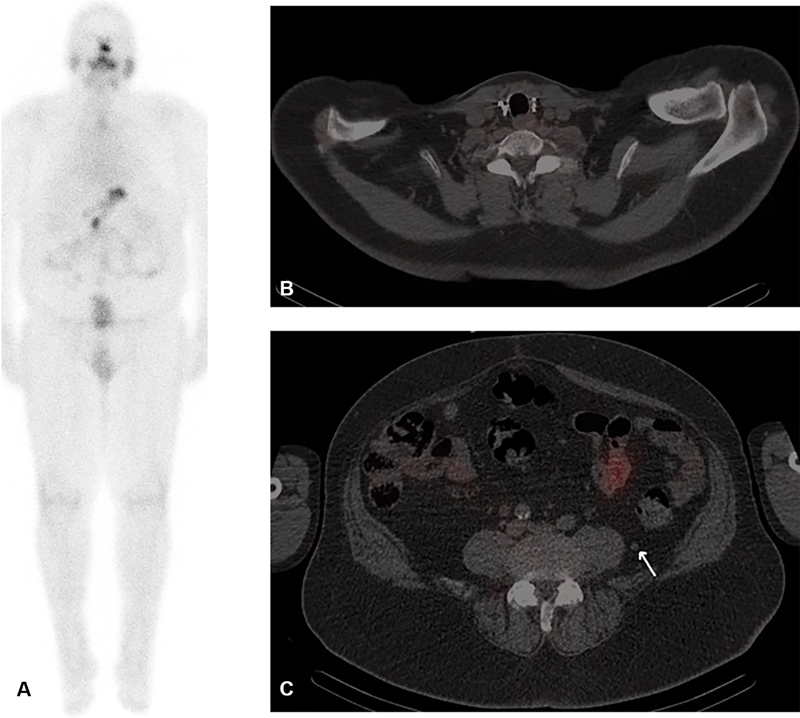
( **A** ) Follow-up ^123^ I single photon emission computed tomography (SPECT)-computed tomography (CT) showing an excellent response to first radioactive iodine treatment (RAIT); ( **B** ) complete ablation of thyroid tissue in the neck; ( **C** ) the left retroperitoneal lesion became significantly smaller and nonavid.

Seven months later, follow-up ^123^ I-SPECT-CT ( Fig. 6 ) showed complete ablation of the thyroid tissue in the neck. The retroperitoneal lesion showed significant reduction from 18 to 7 mm and was no longer iodine avid. However, as small volume disease could not be discounted, follow-up was recommended in 4 months in correlation with thyroglobulin/antithyroglobulin antibodies. Repeat thyroglobulin was persistently negative and thyroglobulin antibodies reduced but were borderline at 4.4 kU/L.

On repeat ^123^ I-SPECT-CTs at 4 and 10 months, the retroperitoneal lesion remained nonavid and became miniscule (5 mm), presumed as fully ablated scar tissue. This indicated an excellent, likely complete response to treatment. As posttreatment thyroglobulin antibodies were borderline, thyroglobulin levels may not be a reliable marker. 4 Therefore, MDM recommended 6 monthly surveillance with ^123^ I-SPECT-CT and thyroglobulin antibody levels for hidden micrometastases and early recurrence.

## Discussion

PSMA expression is seen in the apical membrane and/or cytoplasm. A large study of PSMA expression in different tumors concluded that apical membrane expression is more intense and occurs in PCa while all nonprostatic tumors showed PSMA expression in the cytoplasm, which is less intense. 1 Endothelial PSMA expression can occur in nonprostatic tumor-associated neovasculature (including the nervous system, head and neck, thoracic, abdominal, musculoskeletal, and vascular tumors) despite being absent in benign endothelial tissue. 1 In this case, PSMA-positivity within the retroperitoneal metastasis not only enabled its detection but also assisted in differentiating it from ectopic thyroid tissue, which does not express PSMA.

PSMA expression was present in two-thirds of patients with persistent or recurrent thyroid cancer. 5 High expression was associated with shorter progression-free survival 6 and may be considered a marker of aggressiveness and poorer prognosis. Such tumors could be potential candidates for PSMA-ligand therapy in radioiodine-negative/refractory cases.

Differentiated thyroid cancers (DTCs), comprising of FTC and papillary thyroid cancer (PTC), are usually slow-growing tumors and less likely to metastasize. PTC survival is significantly affected by age, extrathyroid extension, and metastases (typically nodal), while these factors are nonsignificant in FTC, which generally has poorer survival than PTC. 7 Although vascular invasion is characteristic for FTC and therefore distant metastases are more common than in PTC, the management of both PTC and FTC are essentially similar according to current guidelines. 8

Rare sites of distant metastases of FTC in previous case reports include parathyroid, breast, eye (choroid), liver, skin, pleura, heart/pericardium, kidney, ovary, muscle, pancreas, gastrointestinal tract, spleen, parotid, peritoneum, and testes. 9 Some cases of uncommon metastases have been reported years after the diagnosis of DTC. 9 An unusual case of isolated hilar nodal recurrence of PTC was reported in our center 16 years after the diagnosis of the primary. This was incidentally detected as PSMA-positive hilar nodes on a staging PSMA-PET-CT for synchronous PCa. Another case was recently reported in our center where previously treated FTC under evaluation for rising thyroglobulin levels revealed a new renal metastasis on post- ^131^ I RAIT SPECT-CT.

A case series suggested that metastases in unusual sites do not necessarily represent a negative prognostic factor and that if presenting as a single distant lesion, treatment can lead to disease remission 10 as was the outcome in our case.

Despite an extensive review of literature worldwide, there were no previously reported cases of retroperitoneal metastases of thyroid cancer. Hence, this case was the first of its nature, although it is possible that retroperitoneal metastases may have been undetected or inadequately documented in FTC.

The retroperitoneal lesion and the primary FTC were incidentally discovered. Had the patient not been imaged for concurrent PCa (MRI, PSMA-PET-CT), the FTC and metastasis would have remained undiagnosed until disease had progressed further. This case impacts forthcoming clinical practice by stressing the need to further investigate patients with atypical sites of uptake detected on PSMA-PET-CT to exclude synchronous pathologies. PSMA-positive lesion evaluation can be challenging in some instances as illustrated in this case where there was initial difficulty in comprehending the nature of the retroperitoneal lesion. Hence, PET-CT reporters should correlate molecular factors (intensity and extension different from other lesions or from those expected to occur in PCa), morphological factors (CT/MRI findings characteristic of a specific neoplasm or of a PCa lesion), biological factors (known PCa staging, predicted spread, PSA values), and clinical factors (underlying condition that can explain PSMA positivity). 1

This case was discussed in the thyroid cancer MDM, which recommended biopsy of the retroperitoneal nodule, rather than assuming it was related to PCa, which was a crucial decision in the diagnosis of his FTC. The thyroid US and FNAC were reviewed independently at the MDM by a radiologist, pathologist, and members of the multidisciplinary team, leading to both investigations being subsequently upgraded, thus warranting surgery. This highlights the value of multidisciplinary approach in enhancing investigation findings and guiding management.

## Conclusion

DTC may express PSMA leading to pitfalls in scan interpretation. Practitioners should be aware of the spectrum of PSMA expression. These pitfalls can be mitigated through multidisciplinary review or supplementary imaging techniques when unusual distributions of PSMA-positive disease are encountered. This also raises the possibility of diagnostic and therapeutic uses of PSMA-ligand in DTC.

This is the first case described of an unexpected retroperitoneal metastasis from FTC, thus enhancing the understanding of metastatic patterns of DTC. A multidisciplinary approach enabled accurate diagnosis and timely management. The outcome of this case was consistent with a complete response to RAIT, yet continued surveillance is necessary to detect early recurrence in such cases, as PSMA-positivity could indicate more aggressiveness in DTC. The diagnostic intricacies of PSMA-positive findings in nonprostatic malignancies need to be elucidated through further research and case studies in the future.
